# Requesting HIV Results Be Conveyed in-Person: Perspectives of Clinicians and People Recently Diagnosed with HIV

**DOI:** 10.1007/s13178-023-00827-x

**Published:** 2023-06-10

**Authors:** Nathanael Wells, Dean Murphy, Jeanne Ellard, Chris Howard, Phillip Keen, Christopher Fairley, Basil Donovan, Garrett Prestage, on behalf of the RISE Study Team

**Affiliations:** 1 Kirby Institute, University of NSW, Wallace Wurth Building (C27), Cnr High St & Botany St, Kensington, NSW 2052 Australia; 2 Australian Research Centre for Sex, Health, and Society, Bundoora, Australia; 3 Queensland Positive People (QPP), Brisbane, Australia; 4 National Association for People With HIV Australia, Sydney, Australia; 5 Alfred Health, Melbourne Sexual Health Centre, Melbourne, Australia; 6 Central Clinical School, Monash University, Melbourne, Australia

**Keywords:** HIV diagnoses, People living with HIV, PLHIV, HIV management, Phone diagnosis

## Abstract

**Introduction:**

Guidelines recommend that, where possible, clinicians convey HIV-positive test results in person in Australia. However, HIV-negative and all other STI results are routinely delivered by phone or text message. Requesting individuals to obtain positive HIV test results in person could be a deviation from the standard delivery of healthcare and be interpreted as indicating a positive HIV diagnosis.

**Methods:**

This paper is based on two related, ongoing qualitative studies conducted in Australia with HIV healthcare providers and people recently diagnosed with HIV. In study one, in-depth, semi-structured interviews were conducted with people who had recently received a positive HIV diagnosis. In study two, in-depth, semi-structured interviews were conducted with HIV healthcare and peer support providers. Interviews were analyzed thematically.

**Results:**

While clinicians were willing to convey HIV-positive diagnoses by phone, most preferred in-person delivery. In-person delivery enabled clinicians to assess visual cues to better respond to the psychological and emotional needs of patients. For some participants living with HIV, however, the requirement to return to the clinic was interpreted as an unofficial HIV-positive diagnosis. This led to a period in which recently diagnosed participants believed they were HIV-positive without having received an explicit diagnosis.

**Conclusion:**

Protocols for delivering HIV diagnoses by phone, followed by a face-to-face appointment, may reduce the period of anxiety for some patients and assist with an early connection to HIV care and support.

**Policy Implications:**

In some instances, conveying HIV diagnoses by phone may be more appropriate than recalling individuals to the clinic to deliver a positive HIV diagnosis in person.

## Introduction

Australian HIV testing guidelines recommend that, where possible, it is “preferable” to convey an HIV-positive test result in person (ASHM, 2022). The guidelines offer some flexibility, with provisions made for conveying HIV-positive results by phone, text message, email, or videoconference at the discretion of the diagnosing clinician. However, positive and negative sexually transmissible infection (STI) results and negative HIV results are routinely communicated by phone or text messaging services. Requesting positive HIV test results be given in person could be interpreted as unusual as it differs from how other results are conveyed (Grace et al., 2015; Locock et al., 2016). Drawing on accounts of people diagnosed with HIV from 2016 onward and the perspectives of sexual health clinicians, in this paper we explore experiences of and perspectives about asking individuals to return to the clinic to obtain HIV test results.

By reducing HIV viral load to undetectable levels, HIV treatment can minimize the health impacts of HIV, increase the life expectancy of people living with HIV, and eliminate the possibility of onward sexual transmission (Bavinton et al., 2018; Cohen et al., 2016; Rodger et al., 2016). Advances in HIV treatment have led to a reframing of HIV from a likely fatal illness to a chronic, manageable condition (Moyer, 2015; Moyer & Hardon, 2014; Newman et al., 2015). However, receiving an HIV diagnosis remains a significant and challenging experience owing to persistent HIV-related stigma, concerns about the health impacts of HIV, and anxieties of living with a long-term health condition (Pantelic et al., 2019; Sangaramoorthy et al., 2018; Sullivan et al., 2020; Wells et al., 2023). As such, the emotional and psychosocial wellbeing of people living with HIV are crucial considerations when conveying positive HIV diagnoses (Wells et al., 2022).

In a survey of clients of the Sydney Sexual Health Centre, just over half (56%) of respondents indicated a preference for receiving positive HIV diagnoses in person (Martin et al., 2013), while a significant minority (44%) indicated a preference for receiving results remotely (phone, text message, or internet) (Martin et al., 2013). Previous research has also shown that providing people with an option to obtain positive HIV results by phone can increase the likelihood of them receiving their results (McKinstry et al., 2007; Tsu et al., 2002). In a recent analysis, D’Angelo et al. found participants favorable toward receiving HIV diagnoses by phone as it enabled them time to process the diagnosis in familiar settings and at their own pace (D’Angelo et al. 2021). Importantly, despite concerns about the welfare of participants, particularly given the potential distress receiving an HIV diagnosis can cause, no instances of having to engage in crisis services for participants were reported (D’Angelo et al. 2021).

While some research has engaged with perspectives on how HIV diagnoses are conveyed, including the acceptability of conveying HIV diagnoses by phone, our focus here is specifically on the period between being asked to return to the clinic and receiving a formal HIV diagnosis. In this paper, we position the accounts of people who have recently received a positive HIV diagnosis alongside the perspectives of healthcare professionals experienced in conveying HIV diagnoses and providing HIV-related care. In doing so, we draw attention to the way in which current approaches that prioritize conveying HIV diagnoses in person may inadvertently overlook a period in which individuals are in need of, but not engaged with, support services.

### Methods

#### Study Setting

This analysis is based on two related, ongoing qualitative studies in Australia: the first explored the experiences of recently diagnosed people living with HIV, and the second explored how clinicians and peer support workers delivered HIV care in Australia. While conducted separately, contrasting the accounts in both studies enabled consideration of how guidelines were enacted by clinicians and also how they were experienced by people who had received an HIV diagnosis. Both studies were approved by the University of New South Wales Human Research Ethics Committee.

#### Eligibility and Recruitment

Study one: eligible participants were 16 years of age or older, permanent or temporary Australian residents, and had received a positive HIV diagnosis from 2016 onward. Recruitment occurred through sexual health centers, high HIV-caseload private medical clinics, community-based HIV organizations, and/or self-referral. After referral, potential participants were contacted by members of the research team and invited to participate in an interview.

Study two: eligible participants must have had experience in delivering HIV-positive test results and in providing care to people recently diagnosed with HIV in the previous 12 months. Potential participants were identified through purposive sampling methods, contacted via telephone or email by members of the research team, and invited to be interviewed.

#### Data Collection

Study one: semi-structured interviews were conducted with 35 participants who had recently received an HIV diagnosis. Interviews were conducted in person, via telephone, or through video conferencing by members of the research team. Interviews ranged from approximately 30 to 120 minutes in length and were conducted between January 2019 and August 2021. Interviews explored: experiences of diagnosis; HIV treatment and clinical care; engagement with HIV support services; sex and relationships; and the impact of HIV on participants’ lives. Of the 35 participants, 24 completed follow-up interviews after approximately 12 months. Follow-up interviews explored potential changes in participants’ experiences of living with HIV, including treatment, clinical care, access to support services, and sex and relationships. The experience of receiving an HIV diagnosis was not a primary focus of second-round interviews. However, as participants were asked to reflect on the diagnostic encounter, second-round interviews were included as part of the analysis for this article.

Study two: interviews were conducted with ten HIV clinicians and explored approaches to conveying HIV diagnoses, referrals to peer support services, consideration of psychosocial support services, and treatment initiation. Interviews were audio recorded and transcribed verbatim by a professional transcription service. To ensure the confidentiality of both participant cohorts, any potentially identifying information such as names, area of residence, workplace, and/or specific professional role was removed.

#### Analysis

Interview transcripts were entered into NVivo software version 12 and thematically analyzed (Braun et al., 2019). In line with Braun and Clarke's (2021) approach to thematic analysis, our primary aim was not necessarily to reach saturation but, rather, to gain an in-depth understanding of participants’ experiences (Braun & Clarke, 2006). However, upon interviewing 35 participants, it was determined that no new thematic areas were being identified and that effective saturation had been reached (Saunders et al., 2018). Follow-up interviews were conducted to explore any shifts in participants’ experiences of living with HIV, and while participants were asked to reflect on the diagnosis experience, this was not a primary focus. Given the focus of second-round interviews, then, we did not take a longitudinal approach to data analysis (Hermanowicz, 2013). To ensure reliability, members of the research team met regularly to review and discuss findings as they arose. Conceptual and descriptive codes were developed by authors one, two, and three based on a close reading of a small number of interviews and discussions with the larger research team. The analysis for this article was conducted primarily by author one. Drawing on an inductive approach (Boyatzis, 1998) and existing literature, the codebook was refined as new transcripts were analyzed.

#### Findings

Of the 35 participants who had recently received an HIV diagnosis, 24 completed follow-up interviews providing 59 interviews for analysis. Twenty-two participants identified as gay males, eight as bisexual males, three as heterosexual males, and two as heterosexual females. As with clinicians, the experience of being recalled to the clinic to obtain positive HIV test results was not a specific focus of these interviews and emerged in the accounts of the diagnosis experience. As analysis of the data progressed, it became evident that a small subset of participants, mostly gay and bisexual men with previous experience of HIV and STI testing, interpreted the disruption to standard approaches to clinical care as standing in for a formal HIV diagnosis. It is these accounts that are the focus of the following analysis.

The ten clinicians who participated had all delivered at least two HIV diagnoses in the year preceding their interview, and most had several years’ experience in providing HIV care, usually in high HIV-caseload settings. All clinicians had undergone training to prescribe HIV medications and worked in private and/or public health clinics and dedicated sexual health centers. Of the ten clinicians who participated in interviews, seven worked as sexual health physicians, two were general practitioners, and one was a sexual health nurse. While clinicians were not directly asked about conveying a positive HIV test result by phone, most did discuss their attitudes toward how best to deliver HIV diagnoses.

### Being Asked to Return to the Clinic Interpreted as an Informal HIV

#### Diagnosis Disruption to Routine Clinical Care

When describing their experiences of receiving a positive HIV diagnosis, some participants recalled being asked to return to the clinic for their results as marking a difference in their usual experiences of how they received HIV and STI results. Rufus (gay male, 29) stated:

*I got a call off the GP [general practitioner] just to come in to discuss the test results which, as soon as she said that I just knew straight away … I was like, “well, that usually does not happen” (Round [R]1)*

Being asked to return to the clinic differed to how Rufus had previously obtained HIV and STI results. Rufus described a sense of immediately interpreting this request as an unofficial HIV diagnosis. Similarly, Mateus (bisexual male, 29) reflected on being asked to return to the clinic after an HIV test, stating: “*[I’d] had the experience of getting gonorrhoea [previously] so I knew that they text you what you have. And since they didn’t tell me, I was super nervous*” (R1). Mateus continued:

*I was thinking that must have been super-bad because otherwise, they would have just texted me what I had or what happened. And I was scared. So [I got on] my bike and I was really riding super-fast … I wanted to know what was happening. I couldn’t think of anything, I was just rushing to get there (R1).*

Conscious that positive STI results were also communicated via text message, the non-disclosure of results provoked a degree of anxiety over the likelihood of a positive HIV diagnosis.

For some participants, the period in which they believed or suspected a likely positive diagnosis was marked by uncertainty and a feeling of helplessness. Prior to his diagnosis, Oskar (bisexual male, 23) had recently returned from Asia and after feeling unwell for some time, suspected he had some form of tropical infection. Recalling being asked to return to the clinic, Oskar commented:

*The nurse [said] “You need to come in. It’s not urgent but please come in at some point.” I was like: “I’ve either got some very obscure disease or it’s HIV” … I think that’s when it became a kind of live [real] possibility that I was actually going to be diagnosed (R1).*

For Oskar, the experience of being asked to return to the clinic introduced receiving an HIV diagnosis as a “*live*,” or real, possibility. While an HIV test always carries with it the possibility of returning a positive result, this possibility was not one Oskar had initially considered. Rather, it was the request to obtain his results in person that functioned as an indication of a positive HIV test result. Oskar went on to describe the period between being recalled to the clinic and the moment he was told of his HIV diagnosis:

*I was really scared because I didn’t know what to do. There was no real course of action I had. All of this emotion that I wasn’t being provided any sort of avenue to do anything with. I just had to wait … I think that period was just really characterised by fear and impotence (R1).*

For Oskar, this was a period marked not only by fear and anxiety over a possible HIV diagnosis, but also sense of powerlessness and inability to take any meaningful action over his health. With no formal diagnosis, Oskar also felt unable to address his anxieties and fears around a (possible) positive HIV diagnosis.

#### Pre-Test Counselling as Influencing the Likelihood (or Not) of HIV Test Results Being Conveyed by Phone

For Walter (heterosexual male, 28), the interpretation of being asked to return to the clinic as an informal diagnosis occurred precisely because the testing clinician had stated that only HIV-negative test results would be conveyed by phone. Walter recounted the testing clinician stating: “*If the results are negative, the nurse will let you know over the phone. If not, you’ll have to come in and we’ll talk about it*.” Recalling the experience of being asked to return to the clinic, Walter stated:

*The nurse rang on the Thursday and asked if I could come into the clinic and see my GP. As soon as she said that I asked her the question, “Well, can’t you tell me my results?” And she said, “Unfortunately no. I can’t be the one to tell you that. You need to talk to your GP.” As soon as she said that I knew exactly what the diagnosis was and what the truth was.*

Having previously been informed that HIV-positive test results would be conveyed in person, Walter knew to expect that being asked to obtain his HIV test results in person almost certainly indicated a positive test result. While Walter was able to obtain the test results on the same day as being asked to return to the clinic, he nonetheless experienced a period in which he rightly assumed himself to be HIV-positive while not engaged in any form of HIV care.

In the accounts presented so far, it is difficult to ascertain how participants would have reacted to receiving an HIV diagnosis by phone. However, these accounts draw attention to how delaying the delivery of positive HIV test results can function as a diagnosis by proxy. Most participants waited only a short period of time, sometimes less than a day, between being asked to return to the clinic and obtaining a formal diagnosis. Regardless of both the length of time and how requests to return to the clinic were interpreted, participants experienced a period in which they believed themselves to be HIV-positive without being engaged in HIV care or support.

Only one participant in our cohort, Cameron (gay male, 30), had received a positive diagnosis by phone. While traveling interstate, Cameron presented for testing in a regional city and, due to a delay, was scheduled to return home before the results were returned. To avoid further delays, Cameron stated: “*I had made her[physician] promise me that she would tell me over the phone what the result was … I’d rather you tell me than have to go off for another test and then wait*” (R1). Cameron had to negotiate with his testing clinician and stress his preference for receiving results by phone rather than having to undergo another round of tests and having to, once again, wait for the results. Reflecting on his experience of receiving his diagnosis remotely, Cameron commented: “*This doctor was great. She was so good. She called me I think four or five times to check up on how I was going.*” Importantly, the initial testing clinician also provided support by consulting with other clinicians who Cameron subsequently engaged with to manage his health. While Cameron did not receive the *same* form of care as an in-person diagnosis, this did not necessarily translate to inferior forms of healthcare. As he stated, Cameron felt cared for and supported by his clinician, particularly as she kept in regular contact in the period immediately following his diagnosis. Cameron’s account highlights a potential misalignment between what health professionals and policymakers consider as “preferable” when compared with the preferences of some patients.

#### Ensuring Patient Wellbeing at Diagnosis

HIV care providers generally indicated a willingness to convey HIV diagnoses by phone. However, in most cases, this was considered a secondary, less preferable option when compared with delivering HIV diagnoses in person. Justifying this preference, one clinician stated:

*The language tends to be minimal at the point where you say those things [conveying an HIV diagnosis]. I’d say mostly body language and facial expression are the ways that you judge how they’re going with it (Greg, sexual health physician).*

Visual cues such as body language were widely considered a crucial aspect of assessing the psychosocial wellbeing of patients when conveying positive HIV test results (Wells et al., 2022). Greg continued: “it’s very difficult to assess if their mood is okay [by phone] or if … they might want to harm themselves after.” As visual cues could be easily lost when communicating by phone, conveying HIV diagnoses by phone was seen to limit the ability of clinicians to ensure the emotional wellbeing of patients.

Some clinicians were cognisant that by requesting positive HIV test results be conveyed in-person some patients might infer an informal diagnosis. Some felt this even more likely for patients who had previous experience with HIV and STI screening:

*You’ll be phoning them to say that they need to come in to discuss their result rather than getting an SMS saying that their results were negative. And yeah, typically, you’ll feel as though that will lead to a bit of anxiety down the other end of the phone (Colin, general practitioner).*

Despite acknowledging the anxiety that assumptions of positive test results might provoke, this same clinician avoided providing results by phone, even when requested by patients: “Sometimes people will ask to have their results over the phone, but that [giving results by phone] basically never happens.” This account seemingly contradicts that of the previous clinician who expressed that the psychosocial wellbeing of patients when receiving a diagnosis was paramount. Receiving a positive HIV diagnosis can be a difficult experience for many, and recently diagnosed people living with HIV commonly report feelings of shock, distress, and concerns over how HIV might impact their future (Bilardi et al., 2019; van Bilsen et al., 2020). By assuming a positive test result, some patients may experience similar feelings of shock and distress without being engaged in HIV care or support.

Aligning with Australia’s National HIV Testing Policy which includes provision for conveying positive HIV results by phone or video conference (ASHM, 2022), some clinicians indicated a willingness to convey positive results by phone despite their preference for in-person delivery. To determine these circumstances, clinicians often drew on their professional experience and the initial testing encounter:

*[If] they had been in contact with someone with HIV, or there was some discussion around the fact that this test could come back positive – so more extended, pre-test counselling – they’ll usually be called by the nurses or the contacting-tracing people and they can be given the diagnosis over the phone (Greg, sexual health physician)*

For this clinician, the appropriateness of delivering HIV test results by phone was contingent on the likelihood of a positive result and the provision of pre-test counseling. In these circumstances, providing positive results by phone was framed as a standard aspect of their practice. In addition to the initial testing encounter, some clinicians also considered individual patient circumstances important when deciding how best to communicate positive HIV diagnoses:

*Someone who’s got significant mental health issues or significant substance-abuse issues, or substance-abuse disorder, I would … never offer test results by phone. They have to come [in] because they’re a much higher risk than Joe Bloggs of doing something crazy [sic] or just not coming back once they’re phoned (Brendan, sexual health nurse).*

There was a common assumption among clinicians that patients who experience adverse mental health and/or problematic substance use might be less able to cope with an HIV diagnosis and more likely to disengage with clinical care. Conveying positive HIV results in person was therefore considered important to ensure the psychosocial wellbeing of patients and their retention in HIV care.

## Discussion

Aligning with findings from previous studies (Grace et al., 2015), our findings demonstrate that for some participants, requesting HIV test results be obtained in person can be a disruption in standard approaches to the delivery of HIV test results. In Australia, HIV-negative and all STI results are routinely delivered by phone or text message. As some participants (usually GBM who underwent regular HIV and STI screening) had come to expect this process, the delay in providing HIV test results was assumed to indicate a positive HIV diagnosis.

While clinicians in our study generally indicated a willingness to provide HIV diagnoses by phone, in practice, they avoided giving positive HIV results by phone and would instead attempt to convey these results in person. Given clinician participants’ extensive experience in HIV care and their knowledge that receiving a positive HIV diagnosis can be an emotionally challenging and distressing event (Bilardi et al., 2019), their preference for in-person diagnoses was likely motivated by a genuine concern for their patients’ wellbeing. As reported elsewhere (Wells et al., 2022), service providers commonly relied on visual cues such as body language when assessing patient responses to receiving an HIV diagnosis. These cues were felt to be easily lost when conveying positive HIV diagnoses by phone. Moreover, aspects such as pre-existing mental health conditions or substance use also shaped clinicians’ decisions about the appropriateness of conveying HIV diagnoses by phone (Wells et al., 2022).

Current Australian guidelines allow scope for conveying HIV diagnoses by phone and recommend clinicians draw on their expertise when considering how best to convey HIV test results (ASHM, 2022). HIV service providers in our study all had extensive experience in delivering HIV care and diagnoses, worked in high HIV caseload clinics, and were therefore well positioned to draw on their expertise. In Australia, however, increasing numbers of HIV diagnoses are occurring in non-specialist clinics by healthcare professionals with limited experience of providing HIV care (Kirby Institute, 2018). Concrete guidelines on precisely how to convey HIV diagnoses, including by phone where appropriate, might enable less experienced clinicians to better navigate the diagnostic process.

Ensuring people who are newly diagnosed with HIV are engaged in care and initiate treatment as soon as possible after diagnosis is a key aim in the clinical management of HIV and is recognized as a key strategy to reduce rates of new infections (ASHM, 2019; Department of Health, 2018). Asking patients to return to the clinic to obtain HIV results can ensure that people recently diagnosed receive follow-up tests, are provided the relevant information around HIV treatment and care, and are referred to psychosocial and peer support services. Given that conveying results in person can be interpreted as a positive HIV diagnosis, some individuals may experience emotional distress without the support of healthcare professionals. As this study recruited participants who had recently received an HIV diagnosis, assumptions of positive HIV test results were correct. It is possible that in other instances, patients may assume a likely positive result incorrectly. Whether these assumptions are correct or not, causing patients any distress should ideally be avoided. Open discussion of how HIV test results be conveyed, including those that are positive, may facilitate shared decision-making and improve patient healthcare experiences.

Advances in HIV treatment and biomedical prevention strategies have been relatively recent. Until 2011, in Australia, HIV-negative test results were routinely given in person and provided the opportunity to deliver counseling on safer sex practices (Australian Government Department of Health, 2006). Emerging HIV testing technologies, including self- and at-home testing, are shifting the landscape around HIV testing, diagnosis, and HIV-related care (Grace et al., 2015; Lee et al., 2022). These technologies could see more people receiving an HIV diagnosis without the support of healthcare professionals and linkage to care (Lee et al., 2022). Moreover, our study was undertaken prior to the COVID-19 pandemic which saw rapid and substantial changes in healthcare delivery when many clinical consultations were conducted by phone or through video-conferencing software (Thomas et al., 2022). Given our findings that delaying the delivery of test results can be interpreted as an unofficial HIV diagnosis, it is likely this feeling will increase as telehealth becomes more entrenched in the post-pandemic period. Further research is needed to consider both clinician and patient perspectives of HIV diagnoses in the context of these shifts in HIV testing and healthcare delivery.

### Limitations

Our analysis for this paper draws on a subset of participants who were mostly gay and bisexual men. These participants all had previous experiences with sexual healthcare services and were used to receiving HIV-negative test results by phone. It is possible or even likely that those unfamiliar with sexual health services may not interpret the request to return to the clinic to obtain test results as a proxy for an HIV-positive diagnosis. During interviews, participants were not directly asked to reflect on experiences of pre-test counseling, and instead, accounts of pre-test counseling emerged in participants’ accounts of receiving an HIV diagnosis. Moreover, participants living with HIV were also asked to specifically reflect on the test associated with their HIV diagnosis, so their assumptions of a positive diagnosis accurately reflect a positive HIV test result.

Interviews with service providers were conducted as part of a separate sub-study and therefore do not reflect on the same diagnosis encounters as those described by recently diagnosed participants. The accounts presented here, then, do not reflect two perspectives of the same diagnostic encounter. Moreover, the topic of being recalled to the clinic was not a specific focus of interviews, instead emerging through the accounts of participants. Further research that focuses specifically on clinician attitudes toward conveying HIV diagnoses by phone or videoconferencing software is warranted.

## Conclusion

Conveying positive HIV diagnoses can be a complex process, and diagnosing clinicians frequently drew on their own experience when considering how best to deliver positive test results. While delivering positive diagnoses in person is considered preferable, the delaying of results can be interpreted by some as equivalent to a formal diagnosis. Importantly, this can result in some individuals believing themselves to be HIV positive while not engaged in HIV care or receiving emotional support.

### Policy Implications

Requesting individuals return to the clinic to obtain HIV and/or STI results can signal a disruption in standard approaches to sexual healthcare and be interpreted as an informal positive HIV test result. Whether assumptions of a positive test result are correct or not, requesting people to return to the clinic may cause distress and lead to a period in which people believe themselves to be HIV-positive, albeit briefly, but not engaged with HIV healthcare or support services. In some instances, conveying HIV diagnoses by phone may be more appropriate than recalling individuals to the clinic to deliver the news of a positive HIV diagnosis in person. Given previous research has identified acceptability for conveying HIV diagnoses by phone (D’Angelo et al. 2021a), future research could consider this in the Australian context.

## Data Availability

Data for this study is not available due to ethical and privacy concerns.
